# Exsolution on perovskite oxides: morphology and anchorage of nanoparticles

**DOI:** 10.1039/d3cc00456b

**Published:** 2023-03-14

**Authors:** Thomas Ruh, Dominic Berkovec, Florian Schrenk, Christoph Rameshan

**Affiliations:** a Chair of Physical Chemistry, Montanuniversity Leoben 8700 Leoben Austria Christoph.rameshan@unileoben.ac.at; b Institute of Materials Chemistry, TU Wien 1060 Vienna Austria

## Abstract

Perovskites are very promising materials for a wide range of applications (such as catalysis, solid oxide fuel cells…) due to beneficial general properties (*e.g.* stability at high temperatures) and tunability – doping both A- and B-site cations opens the path to a materials design approach that allows specific properties to be finely tuned towards applications. A major asset of perovskites is the ability to form nanoparticles on the surface under certain conditions in a process called “exsolution”. Exsolution leads to the decoration of the material's surface with finely dispersed nanoparticles (which can be metallic or oxidic – depending on the experimental conditions) made from B-site cations of the perovskite lattice (here, doping comes into play, as B-site doping allows control over the constitution of the nanoparticles). In fact, the ability to undergo exsolution is one of the main reasons that perovskites are currently a hot topic of intensive research in catalysis and related fields. Exsolution on perovskites has been heavily researched in the last couple of years: various potential catalysts have been tested with different reactions, the oxide backbone materials and the exsolved nanoparticles have been investigated with a multitude of different methods, and the effect of different exsolution parameters on the resulting nanoparticles has been studied. Despite all this, to our knowledge no comprehensive effort was made so far to evaluate these studies with respect to the effect that the exsolution conditions have on anchorage and morphology of the nanoparticles. Therefore, this highlight aims to provide an overview of nanoparticles exsolved from oxide-based perovskites with a focus on the conditions leading to nanoparticle exsolution.

## Abstract 2

In this work, we summarize the current state of research regarding the morphology of exsolved nanoparticles on perovskite oxides.

## Graphical abstract

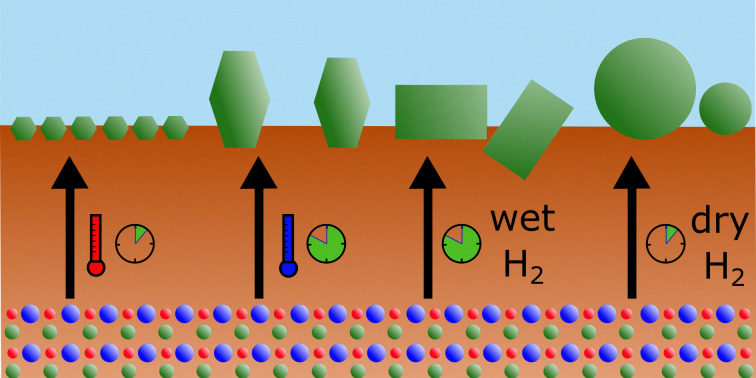

## 1. Introduction

### (a) Perovskites

**Fig. 1:**
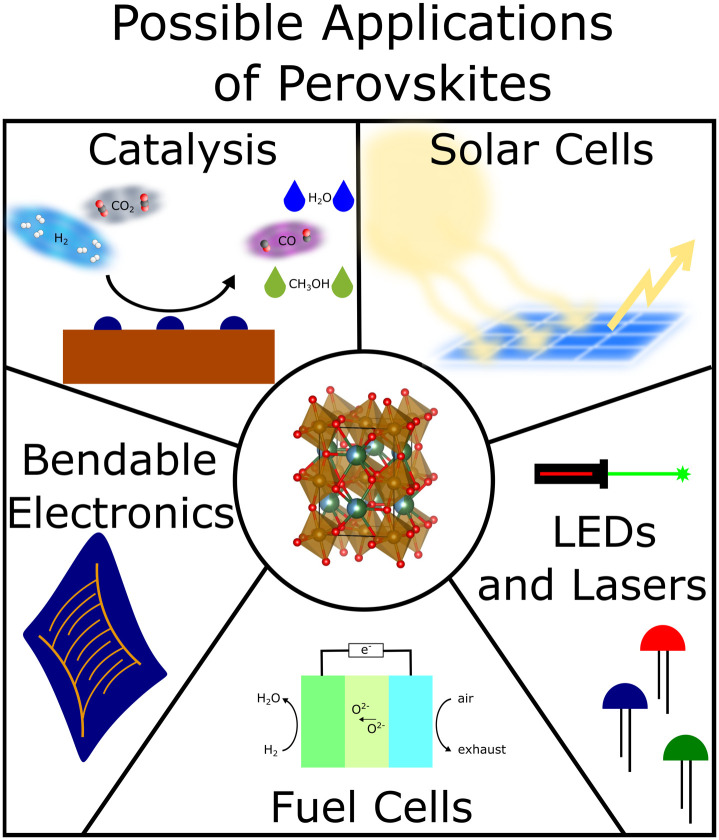
Example applications of perovskites include catalysis,^1–3^ solar cells,^7,8^ LEDs and lasers,^9,10^ fuel cells^4,5^ and bendable electronics.^6^ In the centre, a representation of the unit cell of one of our perovskite oxides is shown.

Perovskites are materials of the general formula ABX_3_, where A and B are different (mostly) metallic cations of differing sizes (A-site cations are larger), whereas X are counter anions. The basic (undistorted) structure is cubic with A cations at the corners. The B cation occupies the centre of the unit cell (*cf.*Fig. 1, middle) and is octahedrally coordinated with X anions – these octahedra form a network with shared corners.

Despite appearing to be deceptively simple materials, perovskites are remarkably compositionally versatile – different A- and B-site elements across the periodic table are possible – and exhibit a vast range of properties that lead to their applicability across many technological fields. To give just a few examples: perovskite oxides have been extensively investigated for applications in catalysis^1–3^ and as electrode materials in solid oxide fuel cells.^4,5^ Perovskite halides are widespread in photovoltaics and solar cells^6–8^ as well as in lasers and light emitting diodes^9,10^ (*cf.*Fig. 1). Perovskite nitrides – albeit not as common – have been predicted to be stable and are expected to be ferroelectric^11^ and more exotic nitrogen-based perovskites have been found to be superconducting.^12^

In our own work, we utilise iron-based perovskite oxides as catalysts for multiple reactions (*e.g.* reverse water–gas shift^13^ or methane dry reforming^14^) because of their highly beneficial properties: this type of perovskite oxides can be used at high temperatures, as they are thermally stable. Due to their compositional flexibility and their tunability (both A- and B-sites can be relatively easily doped with catalytically active elements), perovskite oxides lend themselves to a rational catalyst design approach. Moreover, increased resistance against coking^14,15^ makes these materials interesting for CO_2_ utilization reactions. Another property of these materials – that makes them highly relevant for applications in catalysis and related fields – is their ability to undergo a process called “exsolution” under certain conditions.

### (b) Exsolution

Exsolution as a process has been studied extensively.^13–19^ Depending on the conditions, it reversibly^20^ leads to the decoration of a material's surface with finely dispersed nanoparticles.^16^ The formation of such nanoparticles can be achieved before reactions (in reducing gas atmospheres or electrically by applying a bias to the sample^21,22^) or *in situ*, provided the reaction conditions are sufficiently reducing:^14,23^

**Fig. 2:**
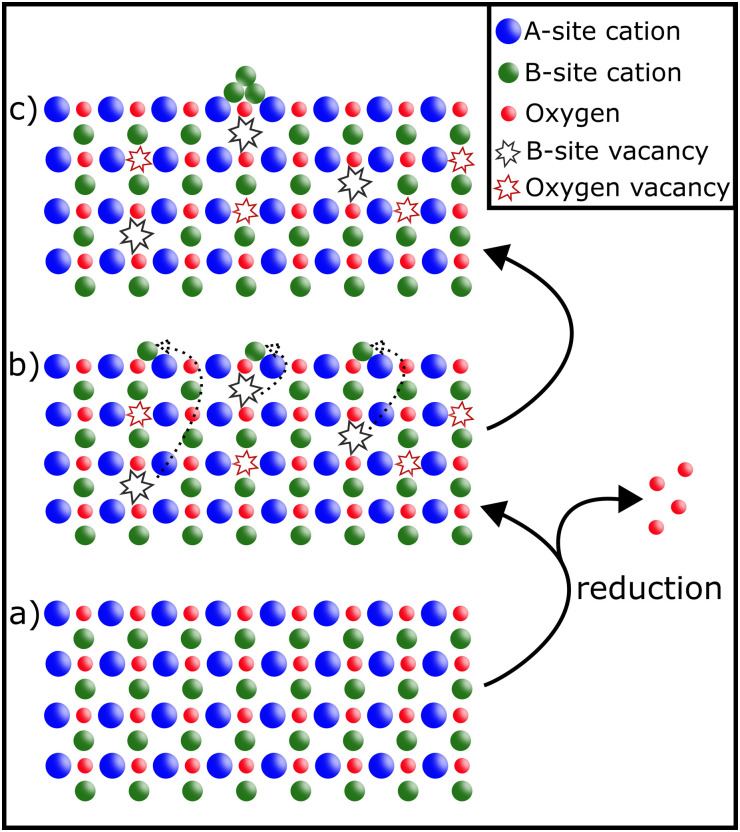
Schematic representation of exsolution: under reducing conditions (a), B-site cations migrate to the surface leaving behind vacancies (b). On the surface they form finely dispersed nanoparticles that are stable against sintering due to them being anchored in the material (c).

The perovskite material is partially reduced causing reducible B-site cations to migrate to the surface, where they exsolve and form metallic or oxidic nanoparticles (depending on the conditions) – as can be seen in Fig. 2.

Among the many advantages of nanoparticles exsolved *via* exsolution are the fine dispersion of the resulting particles,^16^ their high stability and sinter resistance due to “anchoring” in the surface,^15,16,19^ and their tunability: either by varying the composition of the perovskite^16,17,24^ or adapting the conditions during the exsolution process.^18,21,23^ Another feature of exsolution catalysts (provided they exhibit reversible exsolution) is the possible regeneration of the active nanoparticles *via* re-oxidation, thus improving catalyst lifetime.^1,25^

### (c) Scope of this work

Exsolution in perovskite oxides as well as its applications in catalysis and related fields have been the topic of extensive studies, since catalysts with nanoparticles formed *via* exsolution offer a great number of advantages and chances for new and improved catalysts.^13–19^ However, to our knowledge more detailed investigations of the connection between exsolution conditions and morphology (size, population density, shape) and anchorage of the resulting nanoparticles are still rare. Especially so in the context of comparisons across different materials.

With this article, we want to provide a summary of works published on this topic in recent years to serve as starting point for such in-depth comparisons. We strongly believe that it is of vital importance to further deepen the understanding of the relation of exsolution conditions and the properties of the resulting nanoparticles in order to use perovskite oxides (and exsolution) to their fullest potential for the design of new efficient catalysts.

## 2. Overview of published work

For this article, we considered all publications of the Clarivate database “Web of Science”‡‡https://www.webofscience.com by Clarivate Analytics. as well as “Google Scholar”§§https://scholar.google.com. between January 2016 and mid 2022 that contained the keywords “nanoparticle exsolution” and “perovskite”.

**Fig. 3:**
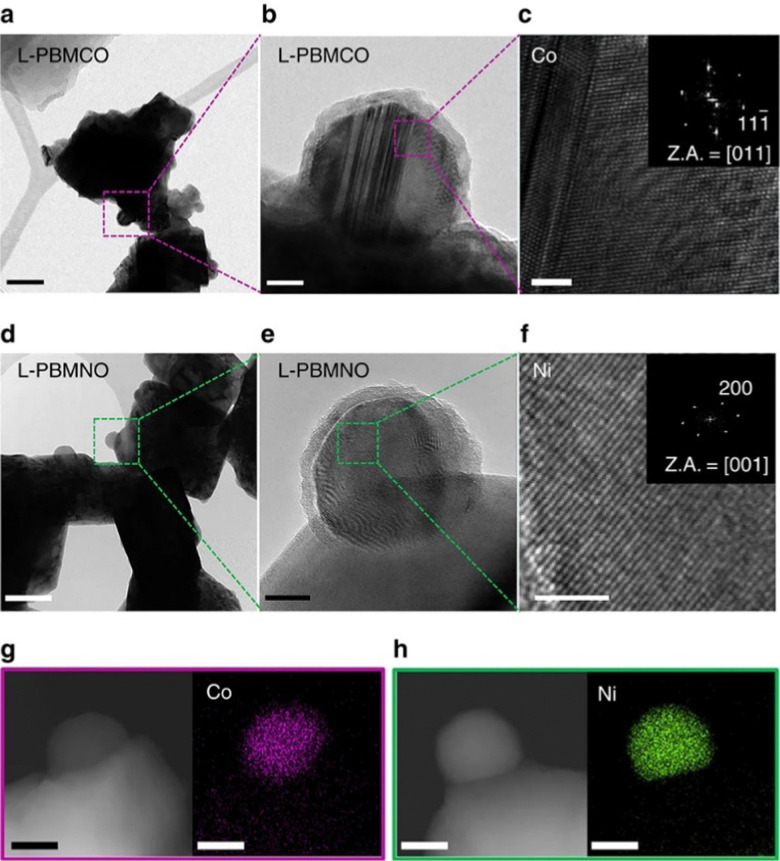
“(a) A bright-field (BF) TEM image; scale bar, 100 nm and (b) high-resolution (HR) TEM image of PrBaMn_1.7_Co_0.3_O_5+*δ*_ (L-PBMCO) sample; scale bar 10 nm. (c) Magnified HR TEM image of exsolved Co nanoparticle; scale bar 2 nm, (d) BF TEM image; scale bar 100 nm and (e) HR TEM image of PrBaMn_1.7_Ni_0.3_O_5+*δ*_ (L-PBMNO) sample; scale bar 10 nm. (f) Magnified HR TEM image of Ni nanoparticle; scale bar 2 nm. (g) High-angle annular dark-field (HAADF) image of the L-PBMCO with the EDS elemental map of Co; scale bar 25 nm. (h) HAADF image of the L-PBMNO with the EDS elemental map of Ni; scale bar 25 nm.” Reproduced from Kwon *et al.*^17^ under CC BY 4.0.

As this overview is focussed on morphology and anchorage of nanoparticle, we further constricted the search to publications containing Scanning Electron Microscopy (SEM) or Transmission Electron Microscopy (TEM) images (ideally both). Both methods are ideally suited to provide information relevant to the question at hand: SEM images not only reveal the surface decoration of the investigated materials – and the fact that exsolution took place, but also allow assessments of size, population density, and shape of the exsolved nanoparticles. TEM images give complementary information about the anchorage of a nanoparticle (in some instances, even the orientation of the visible nanoparticle facet is available). Both microscopy methods additionally offer the possibility to perform elemental analysis to gain information about the make-up of the particles (*via* Energy Dispersive X-ray Spectroscopy – EDX or EDS).^26^ As an example, Fig. 3^17^ shows a series of TEM images that combine anchorage information (the nanoparticles are about 33% embedded in the perovskite surface) with an elemental map revealing the composition of the nanoparticles (shown are Co and Ni nanoparticles, respectively).

By imposing these restrictions, more than 2.500 publications of studies done about nanoparticle exsolution on perovskites could be reduced to about 950 works that also contained at least one SEM/TEM image. To bring this number down even further, publications that did not contain information about the nanoparticle morphology (at least an estimate for the average size or information about the anchorage of the nanoparticles) or that used SEM imaging “merely” as proof for the presence of exsolution were excluded. Furthermore, we eliminated publications with insufficient image quality and/or conclusions we could not reproduce from the presented data.

Ultimately, 83 remaining articles were analysed with respect to the used material (composition of the host material, make-up of the exsolved nanoparticles…), the conditions of the exsolution (pre-treatment/*in situ*, gas atmospheres, temperatures…), and morphology (size, population density, shape) of the exsolved nanoparticles as well as the degree of their anchorage.

### (a) Materials and exsolved nanoparticles

As the publications collected in this work use roughly 125 different perovskite oxide materials, they were grouped by elemental composition of the exsolved nanoparticles (Table 1):

**Table 1:** Elements reported more than once in exsolved nanoparticles

Element	Number of publications	Reported composition of nanoparticles
Ni	42	Ni,^13,17,23,27–47^ Fe–Ni,^35,48–64^ Co–Ni,^65^ Cu–Ni,^66^ Fe–Ni–Re,^49^ Fe–Ni–Mo^58^
Fe	41	Fe,^13,24,67–74^ Fe–Ni,^35,48–64^ Co–Fe,^22,53,75–84^ Fe–Re,^49^ Ru–Fe,^85^ Fe–Ni–Re,^49^ Fe–Ni–Mo^58^
Co	23	Co,^13,17,23,24,40,68,80,86–89^ Co–Fe,^22,53,75–84^ Co–Ni^65^
Ru	6	Ru,^90–94^ Fe–Ru^85^
Ag	4	Ag^95–98^
Cu	3	Cu,^40,99^ Cu–Ni^66^
Re	2	Fe–Re,^49^ Fe–Ni–Re^49^

As Table 1 shows, Ni and Fe are each reported as (part of) exsolved nanoparticles in about 50% of the collected papers, with Co (in slightly more than 25%) a distant third. Ru (6 occurrences), Ag (4), Cu (3), and Re (2) only appear in few publications. Nanoparticles comprised of the following are mentioned once each: Au,^100^ Bi,^70^ Ir,^101^ Mo,^58^ Rh,^102^ (PrBa)O_*x*_,^103^ and SrO.^104^

In terms of mono-metallic nanoparticle composition, Ni particles are reported in 25 publications, whereas Co and Fe are notably less common with 11 and 10 reports, respectively. Fe is the most common element in metal–alloy nanoparticles (34) with Ni (22) and Co (13) far behind. Interestingly, Bi, Mo, and Re are only found in metal–alloy nanoparticles.

Except for two publications, all collected publications report on mono-metallic or metal–alloy nanoparticles, even though (partial) oxidation of the nanoparticles depending on the gas atmosphere during application is conceivable in many cases. Zhu *et al.*^103^ and Ye *et al.*^104^ explicitly study oxidic nanoparticles – (PrBa)O_*x*_ and SrO, respectively.

### (b) Driving force of exsolution

In all of the collected studies – with the exceptions of Fan *et al.*,^22^ where a potential is applied, and Kim *et al.*,^97^ where water mediated exsolution in a H_2_O/O_2_ gas mixture is investigated – reducing gas atmospheres are used to drive exsolution. The most commonly used way to do this, is to use H_2_ in an inert carrier gas (Ar and N_2_ are usually used – refer to Table 2 for the references, however, Kousi *et al.*^36^ use He as inert gas). Alternatives are the usage of wet H_2_ (or “humidified H_2_” with 3% water added) or dry (pure) H_2_. In a few studies, mixtures of reactive gases (H_2_O/H_2_ and H_2_/CO_2_, respectively) in Ar are used to investigate exsolved particles during chemical reactions (*e.g.* during methane dry reforming^49,56^ with addition of CH_4_).

**Table 2:** Gas atmospheres used during exsolution (only the constituent gases are given, and no further distinctions were made regarding other parameters)

Atmosphere	Number of publications	Ref.
H_2_ in Ar	36	28–30, 33, 39, 41, 42, 47–49, 51, 53, 55, 56, 60, 66, 67, 70, 73–75, 78, 80–85, 89, 91–93, 95, 96, 100 and 104
H_2_ in N_2_	20	31, 34, 44, 46, 57, 62–65, 72, 76, 86–88, 94, 95, 98, 99, 101 and 102
wet H_2_	13	17, 24, 27, 30, 35, 38, 52, 54, 61, 68, 69, 77 and 79
dry H_2_	10	37, 40, 43, 45, 58, 59, 81, 87, 103 and 104
H_2_O/H_2_ in Ar	5	28, 32, 50, 71 and 90
H_2_/CO_2_ in Ar	2	13 and 23

Another way of classifying the publications review for this article is to distinguish between exsolution triggered during a pre-treatment before further tests or applications (for instance electrochemical measurements^65^ or NH_3_ sensing^95^) and *in situ* exsolution, *i.e.* nanoparticles are exsolve during tests, applications, or chemical reactions (like the aforementioned methane dry reforming or reverse water–gas shift reaction^13^). Such *in situ* studies are reported in ref. 13, 23, 24, 29, 32, 37, 39, 42, 49, 52, 56, 57, 68, 71, 72, 75, 85, 89, 92, 104.

## 3. Morphology

In this highlight, we focus on three different descriptors of exsolved nanoparticles: (i) the size and/or the size distribution, (ii) the population density and (iii) the shape of the particles. The first two of these can be determined relatively easily during nanoparticle characterisation (*e.g.* by SEM): nanoparticles sizes (of at least one specific type) are given in almost all publications collected for this article (with the exception of 5), whereas the population density is reported in only 22 out of 83 studies. The particle shape, on the other hand, is rarely reported expressly and hardly ever the main topic of investigation (with the notable exception of a study of Kim *et al.*^45^ – see Section 3c). However, it can usually be derived from electron microscope images, given proper image quality.

### (a) Size of nanoparticles

Despite the fact that nanoparticle sizes are reported in almost all publications, comparing the results let alone formulating trends across publications is usually not useful. This is due to the large number of parameters that can be varied during exsolution (temperature, type of atmosphere, pressure, duration, specific composition of the host material…). For instance, the sizes described for Fe nanoparticles (across 7 publications^24,67–69,71,73,74^) span a range from 14 nm up to 600 nm. That is, of course, not surprising given the fact that in those cases 6 different reducing gas atmospheres were used, temperatures between 625 °C and 850 °C were applied and the duration of the reducing treatment varied from 30 minutes to 60 hours.

**Fig. 4:**
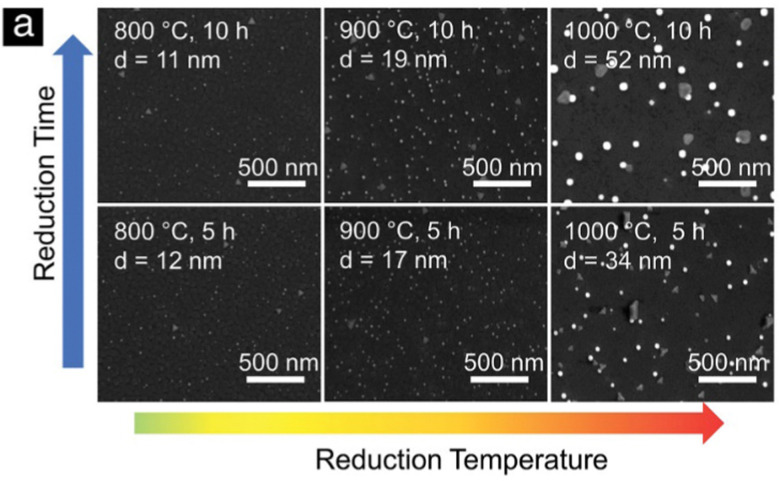
“(2) SEM micrographs of La_0.2_Sr_0.7_Ti_0.9_Ni_0.1_O_3−*δ*_ thin films after reduction at different conditions.” Reprinted (in part) with permission from J. Spring, E. Sediva, Z. D. Hood, J. C. Gonzalez-Rosillo, W. O’Leary, K. J. Kim, A. J. Carrillo and J. L. M. Rupp, *Small*, 2020, **16**, 2003224. Copyright (2020) Wiley-VCH GmbH.

**Fig. 5:**
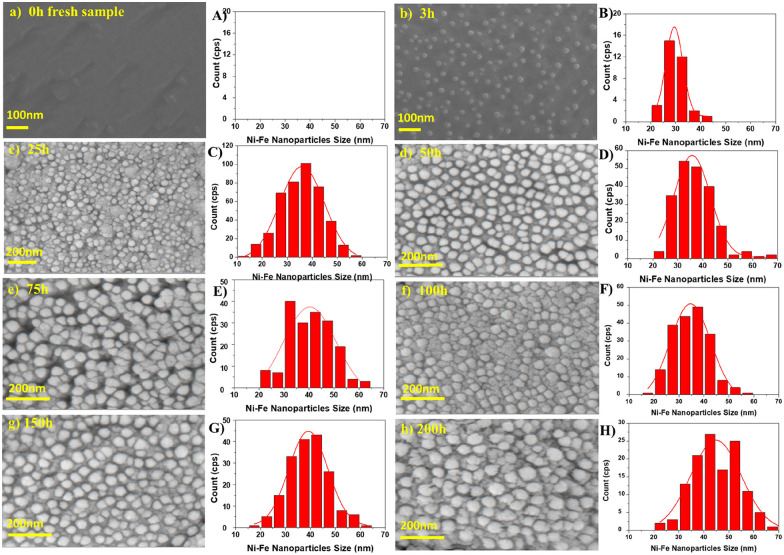
“(a)–(h) SEM images of the surface of *in situ* exsolved Ni–Fe nanoparticle structured SFMNi bar treated at different aging times of (a) 0 h, (b) 3 h, (c) 25 h, (d) 50 h, (e) 75 h, (f) 100 h, (g) 150 h, and (h) 200 h in a condition of 97% H_2_–3% H_2_O atmosphere and 800 °C and (A–H) the particle size distribution and population density of the corresponding *in situ* exsolved Ni–Fe nanoparticles shown in parts a–h calculated by using ImageJ software.” Reprinted with permission from T. H. Zhang, Y. Q. Zhao, X. Y. Zhang, H. Zhang, N. Yu, T. Liu and Y. Wang, *ACS Sustainable Chemistry & Engineering*, 2019, **7**, 17834–17844. Copyright (2019) American Chemical Society.

More meaningful comparisons can be drawn from comparative studies: Spring *et al.*,^39^ for instance, find that Ni nanoparticles grow larger at higher temperatures or if treated for a longer time (see Fig. 4). Islam *et al.*,^65^ Wu *et al.*,^54^ Deka *et al.*^57^ and Cali *et al.*^101^ report the same temperature behaviour of Co–Ni, Fe–Ni, Fe–Ni (albeit on a very small scale), and Ir particles, respectively. Carrillo *et al.*^56^ confirm the same trend for Fe–Ni particles and additionally report broadening of the size distribution. Zhang *et al.*^52^ found similar behaviour when they performed long-term tests of the stability of Ni–Fe alloy particles in an atmosphere of wet H_2_ (see Fig. 5): the particles start small with a narrow size distribution (with an average of 28.9 nm), but after 25 h the average increased to 35.5 nm (and a broader distribution). Subsequent measurements (up to 200 h later) confirmed that nanoparticle size stabilises at this value. Ansari *et al.*^62^ report similar findings for prolonged heat treatment of Ni–Fe alloy nanoparticles (at two different temperatures), where they find a similar initial increase, however, cannot confirm the stabilisation (due to too short measurements).

Horlick *et al.*^58^ investigated the effect of varying substitution (substituting Fe for Ni and Mo on the B-site of SrFeO_3−δ_) and found decreasing size for the alloyed nanoparticles. Wang *et al.*^40^ studied different B-site dopants (Co, Ni, and Cu, respectively) and found different average particle sizes. Sun *et al.*^46^ compared exsolution results of the “conventional” heat treatment of the host material (900 °C for 20 h) with a newly proposed thermal shock method (1400 °C for a few seconds) and report significantly smaller particles.

### (b) Population density of nanoparticles

We will again focus on trends and comparisons found within a given publication (as comparisons across studies are not really meaningful for abovementioned reasons) regarding the population density.

**Fig. 6:**
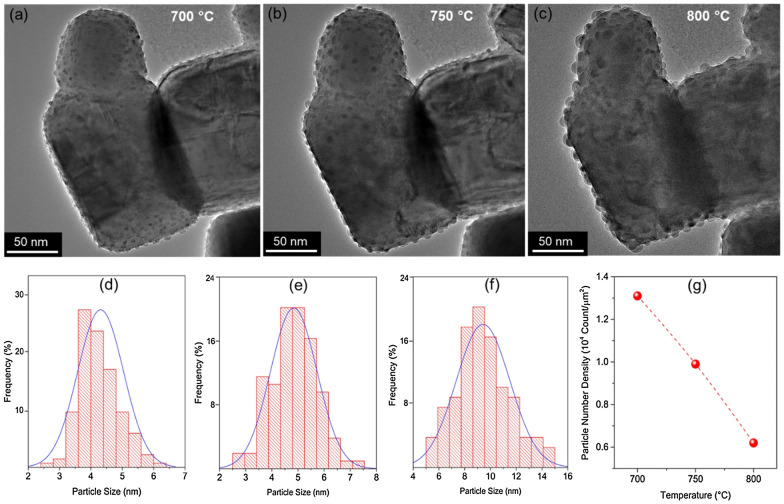
“*in situ* TEM of LSNF under 5% H_2_/N_2_ collected at (a) 700 °C, (b) 750 °C and (c) 800 °C showing formation of nanoparticles, (d)–(f) show distribution of particle sizes at these three temperatures, (g) nanoparticle number density at these three temperatures.” Reprinted from, D. J. Deka, J. Kim, S. Gunduz, M. Aouine, J.-M. M. Millet, A. C. Co, U. S. Ozkan, Investigation of hetero-phases grown *via in situ* exsolution on a Ni-doped (La,Sr)FeO_3_ cathode and the resultant activity enhancement in CO_2_ reduction, *Appl. Catal., B*, **286**, 119917, Copyright (2021), with permission from Elsevier.

In our own work (Lindenthal *et al.*^23^) while performing temperature ramps, we found that the population density of Co nanoparticles tends to decrease with temperature (while the size in this particular case remained rather constant). Deka *et al.*^57^ (see Fig. 6(d)) and Ansari *et al.*^62^ report similar findings for Fe–Ni alloy particles. However, the temperature dependence of the population density seems not as straightforward as was the case for the particle size: Cali *et al.*^101^ and Wu *et al.*^54^ find the opposite behaviour for Ir and Fe–Ni, respectively; with the population density increasing with temperature. Carrillo *et al.*,^56^ on the other hand, find an initial drop of population density for Fe–Ni followed by an increase when increasing the temperature further. Zhang *et al.*^52^ report a similar but inverted trend during sample heat treatment in wet H_2_: they observe a larger number of particles emerging per unit area during the first 150 hours but find a decrease after 200 hours (still at a value above the start).

Another possible factor influencing population density is composition: Horlick *et al.*^58^ observed that during the B-site substitution of SrFeO_3−*δ*_ with equal amounts of Ni and Mo the population density of the alloy particles starts to slowly increase with a notable jump at the highest substitution tested. Wang *et al.*^40^ found different population densities for different B-site dopants (Co, Ni, and Cu, respectively) under otherwise similar conditions. Other factors include applied voltage during electrochemically driven exsolution (Fan *et al.*,^22^ larger voltage leads to increased population density), oxygen partial pressure during water driven exsolution (Kim *et al.*,^97^ higher pressures increase the density), preparation method (Sun *et al.*,^45^ the thermal shock method leads to a significantly larger density), or number of atomic layer deposition cycles (Joo *et al.*^35^ deposit Fe_2_O_3_ on the investigated samples to form Fe–Ni alloy nanoparticles after an additional reduction step and find that more deposition cycles lead to higher particle numbers per unit area, with the particle size, however, remaining rather similar).

### (c) Shape of nanoparticles

Control over the shape of exsolved nanoparticles offers another adjustment screw to optimise the performance of materials or tailor them towards the specific application. For instance, the shape of the catalytically active nanoparticles is crucial in heterogeneous catalysis, where depending on the reaction at hand, the presence of kinks or edges on a non-spherical particle might greatly influence the activity of a given catalyst.^45^

**Fig. 7:**
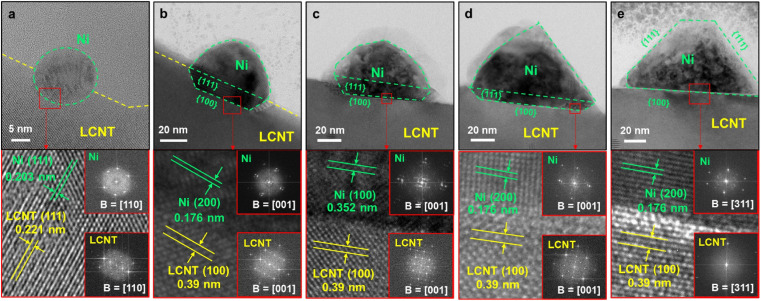
“HVEM images with fast Fourier transform (FFT) of exsolved Ni particles with different size. (a) The spherical nickel nanoparticle on LCNT powder reduced at 800 °C for 24 h, and the polyhedral nickel nanoparticles with different size and shape, on lamella sample from LCNT pellets reduced at 900 °C for (b), (c) 3 and (d), (e) 24 h.” Reprinted from, Y. H. Kim, Y. Kang, H. Jeong, D. Neagu, J.-H. Myung, Shape-shifting nanoparticles on a perovskite oxide for highly stable and active heterogeneous catalysis, *Chem. Eng. J.*, **441**, 136025, Copyright (2022), with permission from Elsevier.

That being said, the work by Kim *et al.*^45^ is the only one of 83 collected works that investigates this possibility in-depth with the goal of designing high performance catalysts. They used a (La,Ca)(Ni,Ti)O_3_ host and investigated exsolved Ni particles, for the exsolution of which variations of reduction temperatures and durations were used. TEM images reveal that depending on the exact conditions different nanoparticles shapes can be found (Fig. 7).

Even though the shape (as well as the size) of a particle can affect the activity and/or the selectivity of a catalyst,^45^ nanoparticle shape appears to mostly be a rather inconsequential descriptor of the exsolved nanoparticles. By far the most commonly observed are spherically shaped particles (42 publications), with ellipsoidal particles reported with only half the frequency of spherical ones (23). Other shapes that occurred during our search are “polyhedral” (2), “pyramid”, “triangular plate”, and “trapezoidal” (1 mention each) – notably, pyramidal, and triangular plate shaped particles only appear in the study by Kim *et al.*^45^ A summary of the reported particle shapes (sorted by shape and grouped by particle composition) is presented in Table 3.

**Table 3:** Overview of reported nanoparticles shapes

Shape	Number of publications	Element/aloy with references
Spherical	42	Ni,^28,31–33,38,39,41,45,47^ Co,^80^ Fe,^67,74^ Ru,^90,93^ Ag,^95–97^ Au,^100^ Fe–Ni,^35,49–51,53–59,62,64^ Co–Ni,^83^ Co–Fe,^75,77,79–84^ Fe–Re,^49^ (Pr,Ba)O_*x*_^103^
Ellipsoidal	23	Ni,^29,30,32,40,46^ Co,^87,88^ Fe,^69,72,73^ Ru,^94^ Ag,^95^ Fe–Ni,^58,60,62–64^ Co–Fe,^53,79,81^ Cu,^40^ Fe–Ni–Re,^49^ (Fe)–Ni–Mo^58^
Polyhydral	2	Ni^45,77^
Pyramid	1	Ni^45^
Triangular	1	Ni^45^
Trapezoidal	1	Ni^78^

## 4. Anchorage

The fact that exsolved nanoparticles are (partially) embedded in the host material is one of their main benefits, as this “anchoring” leads to nanoparticles with higher resistance against sintering compared to other methods.^15,16,19^ This property makes, for instance, catalysts based on perovskite oxides potentially interesting for high-temperature applications, since the catalytically active nanoparticles are anchored and should not be deactivated (as easily).

However, assessing the degree of embedment is often difficult, as one needs good quality TEM images of small areas (like for instance in Fig. 3; whether it is possible to gain information about the degree of anchorage from images of larger areas like in Fig. 6 is open for debate) – *i.e.* it is necessary to find a particle of interest to focus on first. Moreover, this anchorage is often not explicitly reported, but has to be inferred from the images. We summarised the publications that allow the assessment of the degree of anchorage in Table 4.

Studies that allow meaningful comparisons or the formulation of general trends are still rare: for instance, Zhai *et al.*^64^ find similar anchorage (50%) of Fe–Ni nanoparticles independent of reducing atmosphere. Wang *et al.*^40^ report slightly more strongly embedded metal particles when increasing the atomic number of the used B-site dopants (Co, Ni, and Cu, respectively). In Islam *et al.*,^65^ the nanoparticles exsolved at low temperatures sit on top of the surface, while high temperature nanoparticles exhibit anchorage of about 33%. However, whether those findings point to trends or are just anecdotal cannot be assessed at this point, since the number of data points is still far too low.

## 5. Summary and outlook

Nanoparticle exsolution on perovskite oxides has been a “hot topic” and focus of extensive research for more than a decade. Potential materials and applications across many fields – most notably catalysis and electrochemistry (both electrolysis and fuel cells) – are heavily researched to make use of the beneficial properties of materials with nanoparticle surface decoration, where the nanoparticles are produced – either *via* a pre-treatment or *in situ* – by utilising the exsolution capabilities of perovskite oxides.

**Table 4:** Overview of degrees of anchorage

Anchorage	Number of publications	Element/aloy with references
On top (0%)	23	Fe–Ni,^28,37,53,66,71,93^ Ni,^33,45,63,64,97^ Co–Fe,^51,59,83,100^ Co,^45,60^ Fe,^67,86^ Bi–Fe,^52^ Co–Ni,^82^ Fe–Ru,^74^ Ir^99^
25%	8	Ni,^22,45^ Co,^22^ Co–Fe,^73^ Fe,^36^ Fe–Ni,^65^ Fe–Ru,^74^ Ir^99^
33%	16	Co–Fe,^51,60,62,69,70^ Fe–Ni,^32,46,72,85,94^ Ni,^47,83^ Co,^55^ Co–Ni,^83^ SrO^78^
50%	33	Fe–Ni,^37,43,46,50,53,66,89,90,100^ Ni,^24,31,34,41,45,49^ Ag,^38,44,45,84^ Co–Fe,^42,62,69,87^ Ru,^75,83,88^ Fe,^61,91^ Au,^95^ Cu,^22^ Cu–Ni,^13^ Fe–Ni–Re,^46^ (Pr,Ba)O_*x*_^68^

These beneficial properties (such as for instance high thermal stability of iron-based perovskites, sintering resistance of the nanoparticles, and coking resistance of the surface…) have been proven in a vast number of studies: database searches of “nanoparticle exsolution” and “perovskite” yield thousands of results.

It is quite common to characterise the sample(s) with respect to nanoparticle size or degree of anchoring. That being said, population densities of nanoparticle are not as often investigated: almost all studies collected in this work report particle sizes for at least one material, and roughly 80% of papers provide information about anchorage (if not explicitly stated, it is possible to infer from TEM images). Population density, on the other hand, is only addressed in about a quarter of all publications. Moreover, comparisons across different studies are rarely useful, even if data is available, as a large number of adjustable parameters would need to be controlled, which is usually not the case when comparing multiple studies.

However, comparative investigations of the effects of exsolution parameters (temperature, duration, gas atmosphere…) on the nanoparticles (size, population density, anchorage…) are still scarce. Recently, studies have emerged that look into this connection: for instance, Spring *et al.*^39^ showed the influence of reduction temperature and time on the nanoparticle size, Zhang *et al.*^52^ observed nanoparticle size and population density changes over time while exposing their sample to humidified H_2_, Deka *et al.*^57^ compared the effect of different exsolution temperatures on the particles size as well as the population density, and Kim *et al.*^45^ explored how exsolution temperature and duration affect the shape of the resulting nanoparticles.

As we focussed on a general broad overview over recent studies about exsolved nanoparticles, we omitted more in-depth discussions about mechanisms to exsolution itself, particle growth or shape control. Further analyses with respect to these topics can be found, for instance, in ref. 105 and 106.

As stated before, we strongly believe that more comparative studies such as the aforementioned examples are necessary to understand the interplay between perovskite oxide composition as well as exsolution parameter and the formation of nanoparticles. Achieving such understanding is paramount to be able to even better tailor perovskite oxide-based materials toward their desired applications.

Apart from comparative studies, more complete data sets (ideally even under “standardised” testing conditions) would be highly desirable – aside from particle sizes, population densities and shapes, catalytic tests under reaction/application conditions (in case of potential catalyst materials) as well as long-term stability data would be very useful going forward. Moreover, addressing the issue of reversibility of exsolution could be another highly relevant topic of further studies.

We recognize that providing such comprehensive data for every material under investigation can be tedious (especially as materials, exsolution conditions, and applications vary greatly). However, we believe in the vast potential such a collection of data about exsolution materials (a “library of exsolution materials” as it were) could have. Therefore, we sincerely hope that this article may serve as starting point for further and even more in-depth investigations of this matter.

## Author contributions

Conceptualization, C. R.; data curation, T. R. and D. B.; supervision, C. R.; visualization, T. R., D. B. and F. S.; writing – original draft, T. R. and D. B.; writing – review and editing, T. R., F. S. and C. R. All authors have read and agreed to the published version of this manuscript.

## Conflicts of interest

There are no conflicts to declare.

## Supplementary Material
